# Future hydrology and hydrological extremes under climate change in Asian river basins

**DOI:** 10.1038/s41598-021-96656-2

**Published:** 2021-08-24

**Authors:** Sangam Shrestha, Deg-Hyo Bae, Panha Hok, Suwas Ghimire, Yadu Pokhrel

**Affiliations:** 1 Water Engineering and Management, School of Engineering and Technology, Asian Institute of Technology, P.O. Box 4, Klong Luang, Pathum Thani 12120 Thailand; 2 Department of Civil and Environmental Engineering, Sejong University, 98 Gunja-dong, Gwangjin-gu, Seoul, 143-747 Korea; 3 Department of Civil and Environmental Engineering, Michigan State University, East Lansing, MI 48824 USA

**Keywords:** Climate sciences, Environmental sciences, Hydrology

## Abstract

The diverse impacts of anthropogenic climate change in the spatiotemporal distribution of global freshwater are generally addressed through global scale studies, which suffer from uncertainties arising from coarse spatial resolution. Multi-catchment, regional studies provide fine-grained details of these impacts but remain less explored. Here, we present a comprehensive analysis of climate change impacts on the hydrology of 19 river basins from different geographical and climatic conditions in South and Southeast Asia. We find that these two regions will get warmer (1.5 to 7.8 °C) and wetter (− 3.4 to 46.2%) with the expected increment in river flow (− 18.5 to 109%) at the end of the twenty-first century under climate change. An increase in seasonal hydro-climatic extremes in South Asia and the rising intensity of hydro-climatic extremes during only one season in Southeast Asia illustrates high spatiotemporal variability in the impact of climate change and augments the importance of similar studies on a larger scale for broader understanding.

## Introduction

The spatiotemporal variability of global freshwater has been increasing in the past few decades^1–3^. Among several associated reasons, anthropogenic climate change has been identified as a principal driver of such hydrological alteration^4,5^. Previous studies show that streamflow in major rivers across the globe is expected to range from − 96 to 212% within 50 years accompanied by an increase in intensity of hydrological extremes in terms of both magnitude and frequency^1,2,6^. In such a scenario, an understanding of river hydrology and its evolution within future climatic conditions is of immense significance for water resources management and sustainable development. Previous studies show that most climate variables, with the exception of temperature, are expected to experience varying trends in time and space, as does the flow regime^7–11^. However, it is crucial to note that most global scale studies are often conducted at a spatial resolution which is too coarse to capture local climatic phenomena. On the other hand, basin scale studies can capture the local phenomena, but are limited within the watershed scale, failing to provide a holistic picture over a large region. In this context, this study investigates the impact of climate change on the hydrology of 19 river basins (Supplementary Table 5 and Supplementary Fig. 6) in Asia with the aim of capturing trends in relation to different geo-climatic conditions.

The Southern and Southeast Asia region is recognised as being one of the fastest growing in the world, comprising mostly developing countries and catalysing national growth with heavy investment in water infrastructures. Consequently, a clear understanding concerning the variation of future water availability and its spatiotemporal distribution could be a significant asset to the scientific community and policymakers in investigating the associated risks while formulating any kind of river basin management plans and disaster risk reduction (DRR) policies. Thus, this study utilises locally available data in conjunction with a physics-based hydrological model and an ensemble of climate models to provide a reliable outlook at regional level.

Among 19 General Circulation Models (GCMs), an ensemble of five was selected from the Coupled Model Intercomparison Project (CMIP5)^12^ based on their performance evaluation. This was followed by statistical downscaling using quantile mapping and bilinear interpolation to address the methodological variation^13,14^. Finally, bias correction^15,16^ was applied using distribution and empirical-based quantile mapping to obtain the future climate under two different emission scenarios: Representative Concentration Pathways (RCPs) 4.5 and 8.5 in three future periods: the 20 s (2011 to 2040), 40 s (2041 to 2070), and 80 s (2071 to 2100). The results were then compared with those for the control period (1976 to 2005). Thus, climate data obtained was employed in the Soil and Water Assessment Tool (SWAT)^17^ model to execute future hydrological responses. Here, hydrological model performance was evaluated through graphical interpretation and statistical indicators^18^ whereas future hydrological responses were examined through the absolute or percentage change in annual and seasonal flows. Similarly, potential hydrological extremes were evaluated based on streamflow exceeding a given percentage (Q5 and Q95) of time over the analysis period^19^.

## Results

### Changes in future temperature

**Figure 1:**
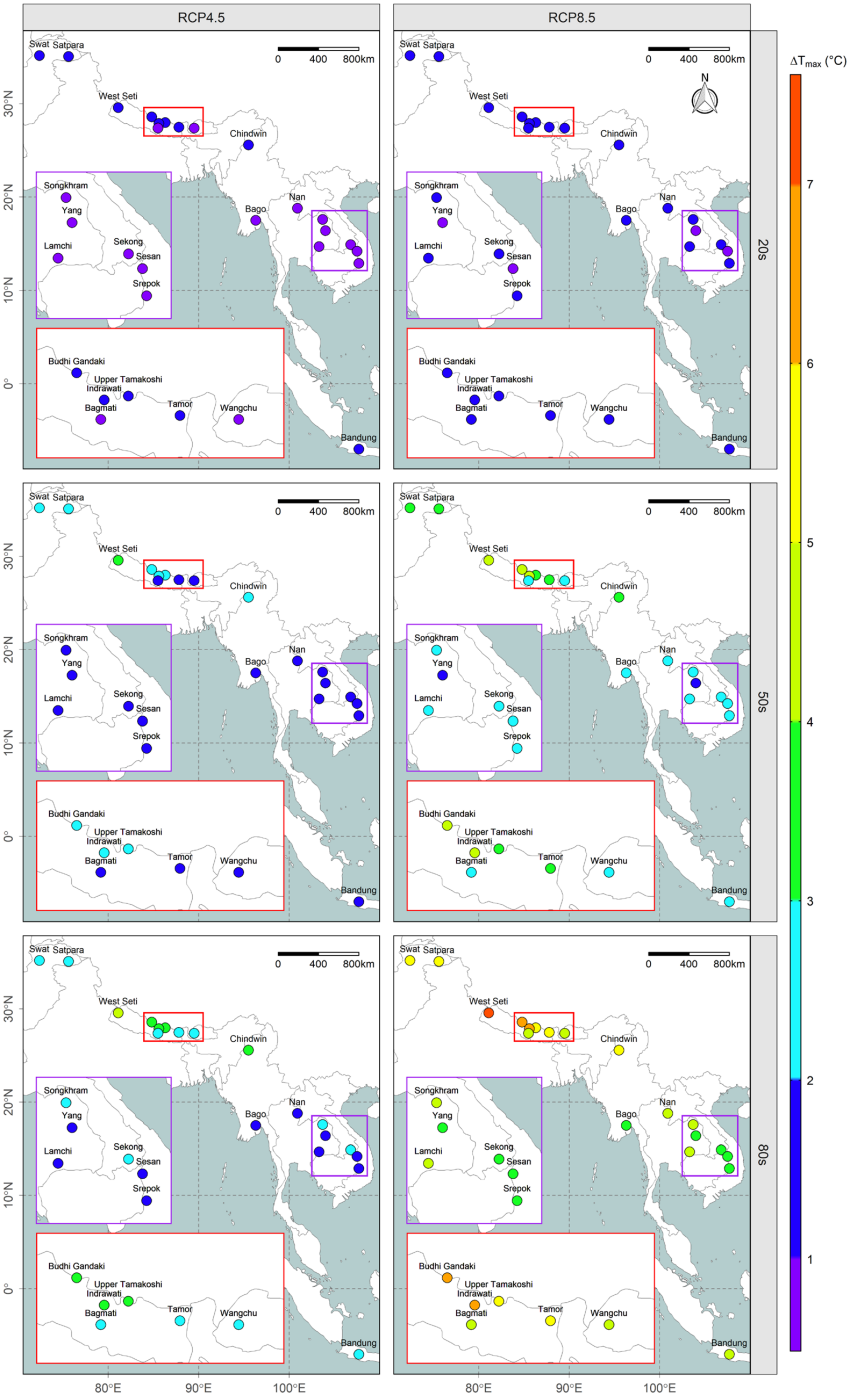
Spatiotemporal variation in the annual maximum temperature anomaly (absolute change). The results are from an ensemble of GCMs, with each facet corresponding to a different emission scenario (RCP4.5 or RCP8.5) during various time periods (20 s, 50 s, or 80 s) as indicated at the top and left side of the map, respectively while the two insets are enlarged and colour-coded^20–22^.

**Figure 2:**
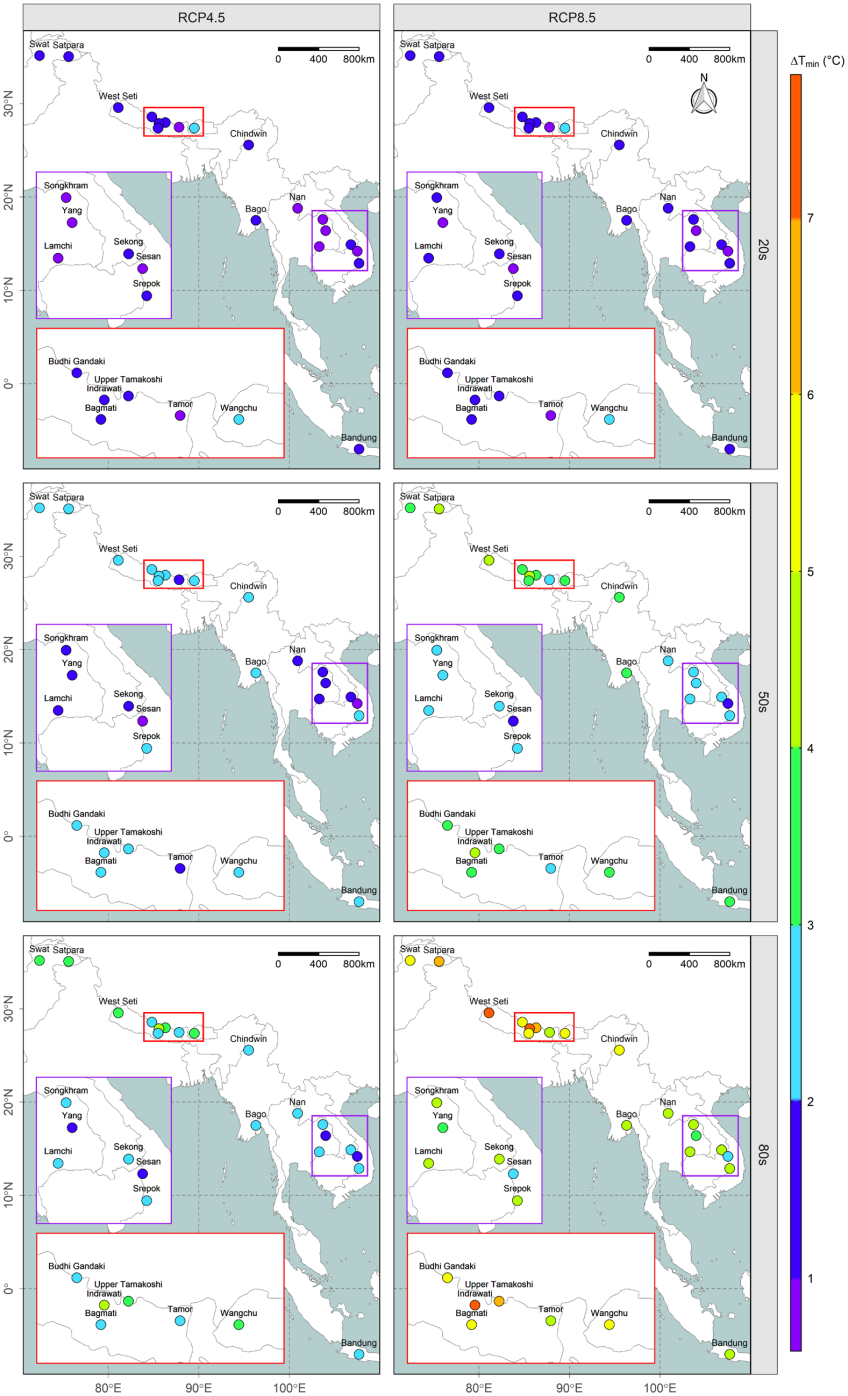
Spatiotemporal variation in the annual minimum temperature anomaly (absolute change). The results are from an ensemble of GCMs, with each facet corresponding to different emission scenario (RCP4.5 or RCP8.5) during various time periods (20 s, 50 s, or 80 s) as indicated at the top and left side of the map, respectively while the two insets are enlarged and colour-coded^20–22^.

An overall increasing trend was found in annual and seasonal maximum temperature over the region (see Fig. 1 and Supplementary Fig. 2). Figure 2 and Supplementary Figure 3 illustrate similar results for minimum temperature. In addition, most of the river basins possess a slightly higher increment in maximum temperature during the months of June, July, and August (JJA) under emission scenario RCP8.5, while no characteristic trend is found in projected temperature across seasons within the river basins. In the South Asian river basins, the annual change in maximum temperature is estimated to remain within the range 1 to 1.5 °C (1.2 to 2 °C), 1.8 to 3.1 °C (2.7 to 4.7 °C), and 2.3 to 4.1 °C (4.5 to 7.6 °C) under RCP4.5 (RCP8.5) in the 20 s, 50 s, and 80 s. Similarly, in the Southeast Asian river basins, the annual change in maximum temperature is estimated to remain within the range 0.5 to 1.4 °C (0.8 to 1.8 °C), 1.1 to 2.6 °C (1.8 to 3.9 °C), and 1.5 to 3.2 °C (3.1 to 5.8 °C) under RCP4.5 (RCP8.5) in the 20 s, 50 s, and 80 s, respectively.

At the end of the twenty-first century, the estimated change in both maximum and minimum annual temperature in the South Asian river basins is expected to remain at around 1 °C higher than for Southeast Asian river basins under both emission scenarios. Although the results for individual basins may vary, Supplementary Figures 2 and 3 clearly show that the South Asian river basins are projected to become much hotter than those of Southeast Asia in future. Moreover, at the end of the century, it is estimated that the change in both maximum and minimum temperature under the higher greenhouse gas (GHG) concentration emission scenario (RCP8.5) is expected to be around 1 °C higher than for the lower GHG concentration emission scenario (RCP4.5) in both South and Southeast Asian river basins. Details of the seasonal variation in maximum and minimum temperature for all river basins are provided in Supplementary Figures 2 and 3.

### Changes in future precipitation

**Figure 3:**
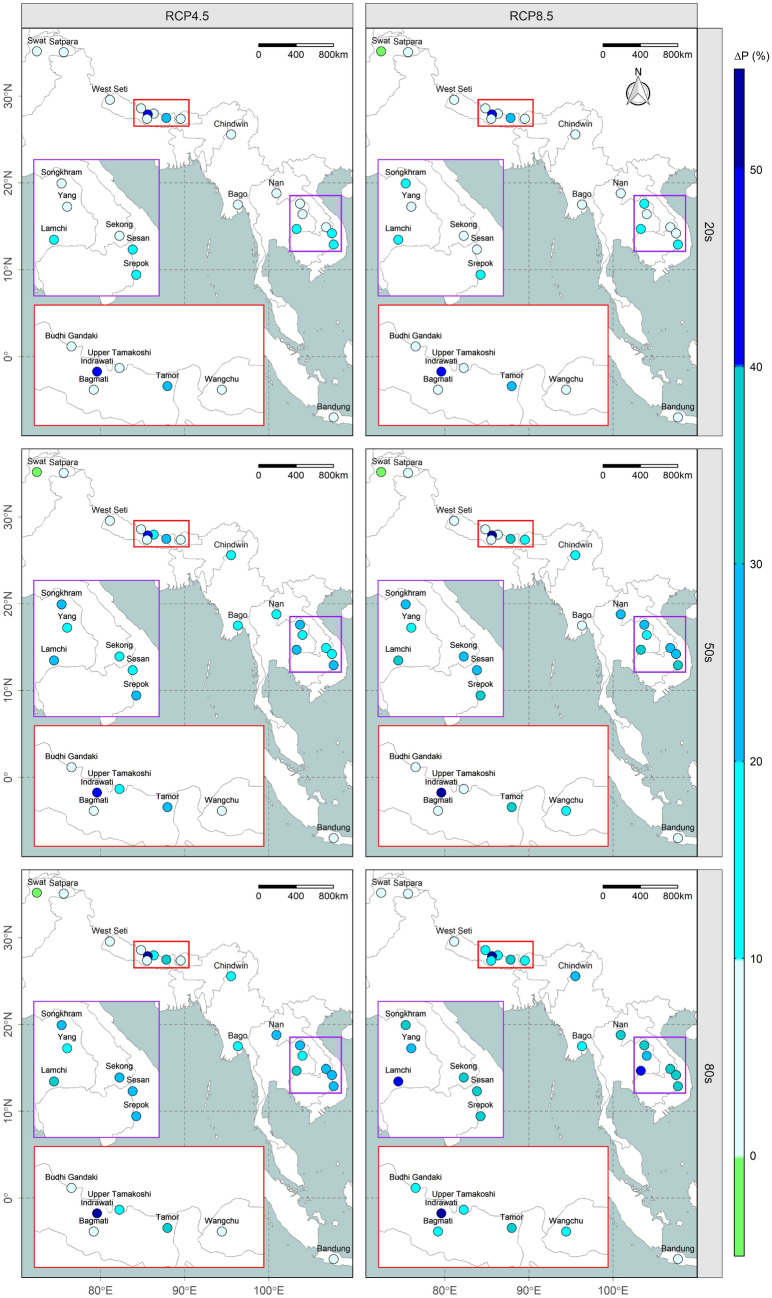
Spatiotemporal variation in the annual precipitation anomaly (% change). The results are from an ensemble of GCMs, with each facet corresponding to different emission scenarios (RCP4.5 or RCP8.5) during various time periods (20 s, 50 s, or 80 s) as indicated at the top and left side of the map, respectively, while the two insets are enlarged and colour-coded^20–22^.

Figure 3 shows an overall increasing trend in annual precipitation over the region except for the Swat River Basin which exhibits a decreasing trend. Interestingly, the change in average annual precipitation in the Southeast Asian river basins is projected to be around twice as much as those in South Asia throughout the century under both emission scenarios. In South Asian river basins, the percentage change in annual precipitation is estimated to remain within the range − 0.5 to 4.9% (− 0.3 to 7.5%), − 4.3 to 12.6% (− 3.2 to 10.7%), and − 3.4 to 16% (1.4 to 18.8%) in the 20 s, 50 s, and 80 s under emission scenario RCP4.5 (RCP8.5). Similarly, the percentage change in annual precipitation within the Southeast Asian river basins is estimated to remain within the range 2.2 to 14.1% (1.4 to 12.3%), 3.4 to 23.6% (6.7 to 31.8%), and 5.4 to 31.5% (7 to 46.2%) in the 20 s, 50 s, and 80 s under emission scenario RCP4.5 (RCP8.5). Considering the seasonal change in precipitation, most of the South Asian river basins show a decreasing trend in the dry period and vice versa except for Swat and Satpara which show contrasting trends (see Supplementary Fig. 4). However, Southeast Asian river basins show an increasing trend in future precipitation throughout the year except Chindwin and Bandung which exhibit similar characteristics to South Asian river basins. Details of the seasonal variation in precipitation for all river basins are provided in Supplementary Figure 3.

### Changes in future streamflow

**Figure 4:**
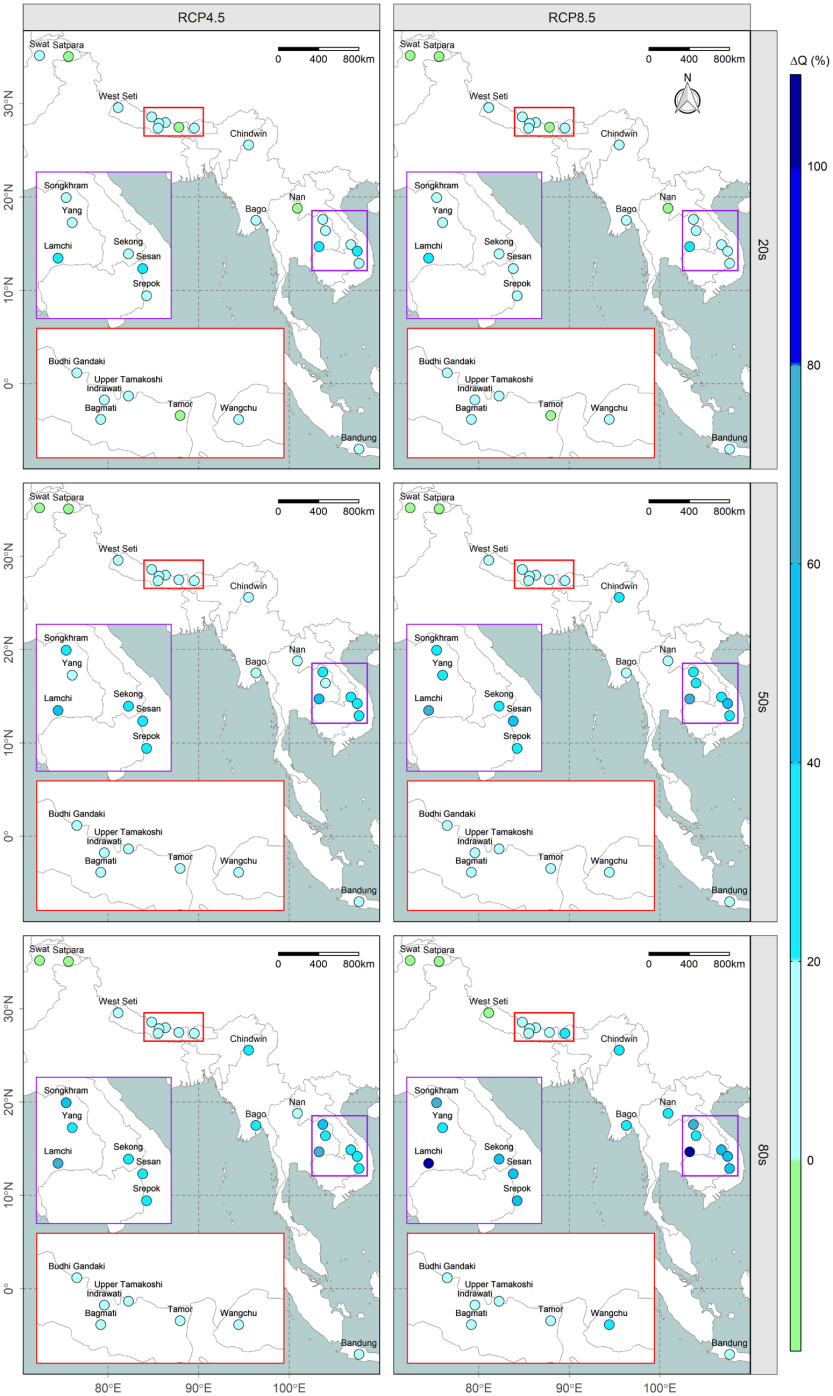
Spatiotemporal variation in the annual river discharge anomaly (% change). The results are from ensemble of GCMs, and each facet corresponds to different emission scenarios (RCP4.5 or RCP8.5) during different time periods (20 s, 50 s, or 80 s) as indicated at the top and left side of the map, respectively while the two insets are enlarged and colour-coded^20–22^.

Figure 4 illustrates an overall increasing trend in annual flows for most of the river basins with a few exceptions like Swat and Satpara which show a decreasing trend. The projected annual river discharge in Southeast Asian river basins is also prominently higher, (around five times) than in South Asian river basins. In South Asian river basins, the percentage change in annual discharge is estimated to range from − 2.8 to 6.6% (− 7.6 to 8.5%), − 12.3 to 15% (− 19 to 14.2%), and − 14 to 18.8% (− 18.5 to 26.3%) in the 20 s, 50 s, and 80 s under emission scenarios RCP4.5 (RCP8.5). Similarly, the annual percentage change in discharge within the Southeast Asian river basins is estimated to range from − 0.9 to 25.1% (− 0.7 to 27.6%), 2.1 to 53.2% (6.8 to 70.3%), and 4.9 to 71.9% (6.3 to 109%) in the 20 s, 50 s, and 80 s under emission scenario RCP4.5 (RCP8.5). Analysis shows that most of the river basins in the region are likely to demonstrate an increasing trend in seasonal flow with few exceptions. Any decrement in projected flow is mainly expected to occur during the drier period of the year. Although both regions reveal similar results, fewer basins in Southeast Asia demonstrate a decreasing trend under seasonal flow. Details of the seasonal variation in flow for all river basins are provided in Supplementary Figure 5.

### Changes in future hydrological extremes

**Figure 5:**
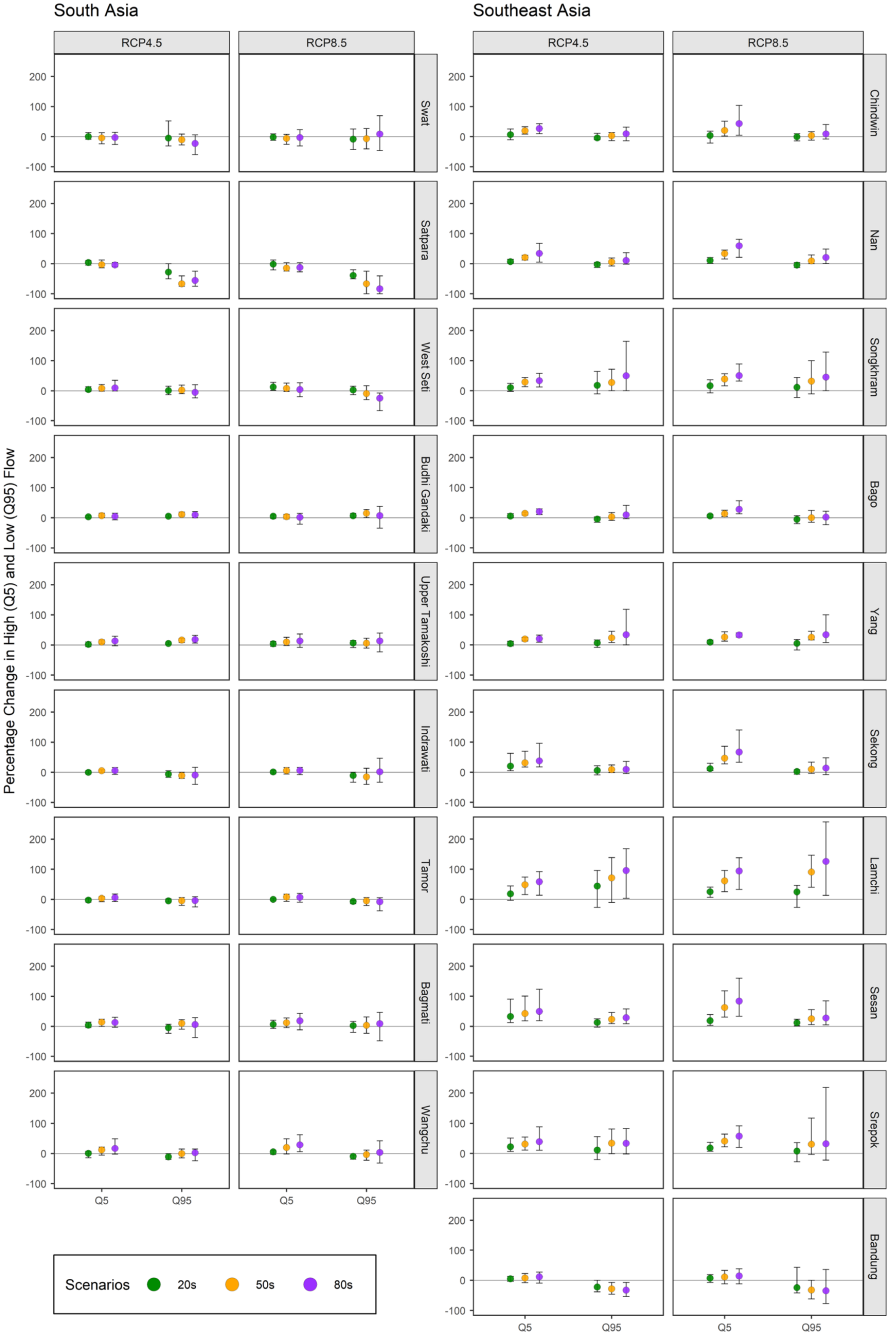
High (Q5) and low flow (Q95) anomaly (% change) in South Asian and Southeast Asian river basins. Each facet corresponds to a different emission scenario (RCP4.5 or RCP8.5) in different basins as indicated at the top and left side of the chart, respectively. The error bar represents the minimum and maximum value among GCMs while the point represents the mean value^20,22^.

Figure 5 portrays an overall increasing trend in high flow for most river basins under study with a few exceptions like Swat and Satpara which show a decreasing trend. The expected increment in high flow for Southeast Asian river basins is also around four times higher than in South Asian river basins. The percentage change in high flow within South Asian river basins is estimated to range from − 2.7 to 4.2% (− 2 to 12.6%), − 4.2 to 14% (− 14.4 to 19.8%), and − 4 to 16.9% (− 12.5 to 28.5%) in 20 s, 50 s, and 80 s under emission scenario RCP4.5 (RCP8.5). Similarly, the percentage change in high flow within the Southeast Asian river basins is estimated to range from 4.2 to 32.8% (3.4 to 24.8%), 7.2 to 48.3% (11 to 62.6%), and 11.3 to 58.3% (14.4 to 93.9%) in the 20 s, 50 s, and 80 s under emission scenario RCP4.5 (RCP8.5).

On the other hand, the future projection of low flow shows a mixed trend within South Asian river basins, whereas it tends to rise in Southeast Asian river basins with a few exceptions, such as the Bandung River Basin in Indonesia which exhibits a decreasing trend. The increment in low flow is mainly expected to be higher than for high flow within Southeast Asian river basins. The percentage change in low flow within South Asian river basins is estimated to range from − 27.8 to 5% (− 38.9 to 6.5%), − 66.7 to 16.1% (− 66.7 to 14.8%), and − 55.6 to 18.4% (− 83.3 to 13.1%) in 20 s, 50 s, and 80 s under emission scenario RCP4.5 (RCP8.5). In contrast, the percentage change in low flow within the Southeast Asian river basins is estimated to range from − 22.6 to 44% (− 24.5 to 24.7%), − 28.3 to 71.3% (− 32.1 to 90.7%), and − 33 to 95.3% (− 34.9 to 126%) in the 20 s, 50 s, and 80 s under emission scenario RCP4.5 (RCP8.5).

### Uncertainty and implication of the result

Uncertainties in modelling studies are inherent. The major sources of uncertainty in this study can be attributed to climate modelling, downscaling and hydrological modelling where, projection of climate variables have uncertainty from the climate modelling and downscaling only while projection of the discharge has hydrological model uncertainty added on top of it. Since the objective of the study is not to quantify each sources of uncertainty rather have general understanding the level of uncertainty within the result, we tried to explore the possible range of variation within the result as portrayed in Supplementary Figure 2, 3, 4 and 5 using error bars. We found that the range of projection of future maximum temperature was higher in case of South Asian basins than the Southeast Asian basins. Notably, Tamor, West Seti and Budhi Gandaki have the wider range of projection than other basins. Also, the range of projection is wider in RCP8.5 emission scenario than the RCP4.5 in both regions. Similar result is found in case of minimum temperature however, the highest range of variation is in West Seti, Upper Tamakoshi and Budhi Gandaki river basins. Since temperature is much sensitive to the physiography of the location such a result must be due to extreme variation in topography of the selected South Asian basins compared to Southeast Asian basins. On the other hand, uncertainty in future precipitation is found higher in Southeast Asian basins than the South Asian basins. Southeast Asian basins have highest range of percentage variation in dry season precipitation which do not contribute much to the overall annual precipitation given that the dry season precipitation for the given basins: Bago, Nan, Songkhram, Lamchi, Sesan and Sekong are the lowest among all during the baseline period. The projection of future discharge using climate variables from GCMs show higher range of variation within Southeast Asian basins than South Asian basins except few cases like Swat and Satpara basin. This is likely possible owing to high range of uncertainty in precipitation within Southeast Asian basins and added uncertainty from hydrological modelling. In addition, basins like Swat and Satpara followed by Lamchi, Wangchu, Nan, Srepok and Bago have high bias during model validation attributed to effect from reservoir, short duration of data and sparsely distributed climate data.

Accounting all these uncertainties, the implication of climate change in most of the basins are still pronounced in terms of temperature with a clear increasing trend, given that the projected change in mean annual maximum and minimum temperature between 0.5 to 7.6 °C are still on the higher end as compared to the projection by IPCC^23^. In context of Himalayan basins, mostly from South Asia it could have serious impact due to the presence of several glacier lakes which could eventually cause Glacier Lake Outburst Floods (GLOFs). In Southeast Asian region such an increase in temperature would create unbearable environment for living and increase the risk of drought. Similarly, temporal changes in precipitation pattern such as high increase in dry season precipitation are likely to change annual crop cycle. Since most of the basins from Southeast Asia are agricultural basins, the impact is anticipated to be more significant in the region than in South Asia. In addition, the impact is carried out in the flow regime with high dry season flow which is likely to impact the aquatic habitat since the lowest flow is one of the important phases to sustain the riverine ecosystem. Chindwin, Nan, Bago and Bandung have considerably small change in the flow while other basins from Southeast Asia have significant increase in discharge thus increasing the flood risk. In South Asian region change in flow regime is considerably lower than Southeast Asia. However, all the basins except Swat and Satpara have clear increasing trend in annual discharge. Given the fact, flow in Swat and Satpara are much affected by reservoir and data scarcity with a high error in water balance estimation further research is felt necessary for better understanding of these basin hydrology.

Hydrological responses from most of the basins are found to be closely related to their geography and climatic conditions. Owing to the snow-fed origin and orographic rainfall pattern, most of the South Asian basins’ hydrology are found to be less sensitive towards the climate change than Southeast Asian river basins where basins’ hydrological responses are highly sensitive towards prevalent convective rainfall patterns. Moreover, Chindwin and Bandung river basins possess much similar hydrological responses to South Asian river basins as their geography and climatic conditions are similar to South Asian river basins. The resemblance in precipitation pattern with South Asian river basins also strengthen the fact that the evolution of future climate and basin hydrology is much dependent on their present climate and geographic condition despite their location.

## Discussion

It can be concluded that both South and Southeast Asian river basins are likely to be warmer in the future, with the South Asian region becoming more than 1 °C hotter than the Southeast Asian region under both emission scenarios (i.e., RCP4.5 and RCP8.5). Furthermore, the increment in temperature is expected to be more than 1 °C higher under RCP8.5 than RCP4.5. In the future, most South Asian river basins are likely to become drier during the dry season and wetter during the wet season, except for Swat and Satpara which exhibit contrasting results. Whereas Southeast Asian basins are expected to become much wetter in the future throughout the year except basins like Chindwin and Bandung which show more of a resemblance to South Asian basins. Future discharge projection is coherent to future projections of precipitation, although instances of decreasing streamflow during the dry period of the year are expected in a small number of basins. Analysis of extreme flow suggests that both regions have a tendency towards increasing high flow with a few exceptions such as Swat and Satpara. Whereas South Asian basins show a mixed trend in low flow with most basins likely to follow a decreasing trend while Southeast Asian basins follow an increasing trend in low flow too, except for the Bandung River Basin in Indonesia.

Since this study is not free from uncertainties concerning input, model structure, and initial boundary conditions within the model, we attempt to develop insights based on the patterns found within the projected climate variables and hydrology rather than focusing on precision in estimation. Here, Chindwin and Bandung basins are expected to feature most of the future hydro-climatic conditions of the South Asian region, and this can be associated to their similar present geo-climatic conditions with South Asian River basins. Furthermore, climate change is likely to impact differently on the two regions, with the South Asian region expected to face increased severity during both dry and wet seasons while increased severity mostly in wet season is anticipated for the Southeast Asian region but with much higher intensity. This amplifies the increased risk of water-induced hazard and complications in water resources management within the region. In addition, it also illustrates spatiotemporal variability in the impact of climate change, augmenting the importance of similar studies on a larger scale for broader understanding.

## Methods

A concise schematic representation of the overall research methodology is illustrated in Supplementary Figure 7. Different datasets (hydro-meteorological, geospatial, and GCM) collected from the various sources are first examined for quality and then processed for further utilisation. This includes testing for consistency, missing data imputation, outlier detection and removal, downscaling, bias correction, etc. Thus, processed geospatial and meteorological data is fed into the SWAT model and calibrated with the observed hydrological data using “SWAT-CUP”. The calibrated model is further validated using different time series of hydrological data prior to the hydrological simulation of three future periods: near future as the 20 s (2011 to 2040), mid future as the 50 s (2041 to 2070), and far future as the 80 s (2071 to 2100), with respect to the baseline period (1976 to 2005).

### Study river basins

The study area includes nine river basins in South Asia and ten in Southeast Asia (see Supplementary Fig. 6 and Supplementary Table 5) extending from − 7° 00′ N to 35° 12′ N latitudes and 72° 24′ E to 107° 42′ E longitudes, within nine different countries: Pakistan, Nepal, Bhutan, Myanmar, Thailand, Cambodia, Lao PDR, Vietnam, and Indonesia. The climate in the area varies from hot and humid tropical/sub-tropical to the chill cold arid/semi-arid tundra of the high Himalayas. River basins in South Asia are mainly characterised by mountainous terrain under sub-tropical and tundra climate. In contrast, river basins in Southeast Asia are located in low flatlands and hilly terrain under tropical climate except for the Chindwin River Basin in Myanmar, which possesses similar characteristics to South Asian river basins.

Physiographically, the study area features an extreme elevation range from the lowlands (− 8 m above sea level (masl)) of the Bago River Basin in Myanmar to the high Himalayas (8385 masl) of the Tamor River Basin in Nepal. Similarly, the distribution of drainage area varies widely from 284 km^2^ (Satpara, Pakistan) to 69,924 km^2^ (Chindwin, Myanmar). Monsoons within the region can be distinguished into the dominant southwest summer monsoon from June to September, and the northeast winter monsoon from December to March. The average annual precipitation fluctuates from 418 mm (Satpara, Pakistan) to 2870 mm (Bago, Myanmar), whereas the average annual temperature ranges from − 3 °C (Satpara, Pakistan) to 28 °C (Lamchi, Thailand).

### Data

Table 1 presents details of the data utilised during this research. Global scale geospatial data such as digital elevation model (DEM), soil map, and land cover map are utilised unless available at regional scale, whereas hydro-meteorological data is acquired from the relevant national authorities. Moreover, data from five general circulation models (GCMs) under two emission scenarios RCPs 4.5 and 8.5 is provided by Sejong University to assess the impact of climate change, with all data being thoroughly examined for quality control. The consistency of the precipitation data over the study period is assured through double mass curve analysis, while Asian Precipitation—Highly-Resolved Observational Data Integration Towards Evaluation (APHRODITE) data is utilised for missing data imputation.

**Table 1:** Data and corresponding source.

SN	Data	Time period/frequency	Source/developer
1	Topography		
	ASTER (30 m × 30 m) SRTM (90 m × 90 m)	2000–2013 2000	https://earthexplorer.usgs.gov/ https://srtm.csi.cgiar.org
2	Land cover map		
	ESA (300 × 300) MRC (250 × 250) LDD (Vector data)	1992–2012 2003 2002–2007	https://maps.elie.ucl.ac.be/CCI/ MRC LDD
3	Soil map		
	SOTER (1:1,000,000) MRC (250 × 250) FAO (1:5,000,000)	1980–1990 2003 1971–1981	https://www.isric.org/explore/soter MRC http://www.fao.org/soils-portal/data-hub/en/
4	Hydro-meteorological data		
	Precipitation Temperature Discharge	1979–2016/Daily 1979–2016/Daily 1984–2016/Daily	MRC Relevant national authorities
5	GCMs data RCP4.5 and RCP8.5		
	BCC-bcc-csm1-1-m (BCC-CSM1.1(m))	1974–2100/Daily	BCC, China
	CCCma-CanESM2 (CanESM2)	1974–2100/Daily	CCCma, Canada
	CMCC-CMCC-CMS (CMCC-CMS)	1974–2100/Daily	CMCC, Italy
	CNRM-CERFACS-CNRM-CM5 (CNRM-CM5)	1974–2100/Daily	CNRM-CERFACS, France
	NCC-NorESM1-M (NorESM1-M)	1974–2100/Daily	NCC, Norway

ASTER GDEM, Advanced Spaceborne Thermal Emission and Reflection Radiometer Global Digital Elevation Model; SRTM, Shuttle Radar Topography Mission; ESA, European Space Agency; MRC, Mekong River Commission; LDD, Land Development Department, Thailand; SOTER, Soil and Terrain; BCC, Beijing Climate Center; CCCma, Canadian Centre for Climate Modelling and Analysis; CMCC, Centro Euromediterraneo sui Cambiamenti Climatici; CNRM-CERFACS, Centre National de Recherches Météorologiques—Centre Européen de Recherche et de Formation Avancée en Calcul Scientifique; NCC, Norwegian Climate Centre; RCP, Representative Concentration Pathway. Time period of hydro-meteorological variables is the total range from all basins (detailed in Supplementary Table 6).

### Climate change projection

General circulation models (GCMs) are based upon Navier–Stokes equations on a rotating sphere to simulate the earth’s atmosphere and oceans, and not free from uncertainty due to internal climate variability and errors in the representation of the earth’s system process^24,25^. In order to address such uncertainty, an ensemble of five selected GCMs with two emission scenarios: RCPs 4.5 and 8.5 are used such that the possible range of climate change likely to occur in future can be captured well. The selection of GCMs is made based upon the performance evaluation of climate simulation from 19 GCMs with CMIP5 over the control period (1976 to 2005). The simulation performance of each GCM is statistically evaluated by comparing the meteorological data generated from GCMs with the observation or reanalysis data, and ranking it using the scoring system as shown in Supplementary Table 1.

Owing to their coarser spatial resolution, GCMs are unlikely to represent regional climate constituents such as topography, vegetation, and large water bodies thus compelling the employment of an appropriate downscaling technique^26^. Among two downscaling approaches, namely statistical and dynamical, the former is based on statistical relationships between the meso-scale variables/predictors (e.g., mean sea level pressure, geo-potential height, surface humidity, mean temperature, etc.) and local climate variables/predictands (e.g., precipitation, temperature and potential evaporation)^13,27^, while the latter implies the application of RCMs embedded within GCMs. Due to the application of boundary conditions from the GCM, RCMs are susceptible to the accuracy of their driving GCM. On the other hand, the statistical downscaling approach is subject to uncertainty in future climate relationships between circulation patterns and local climate variables. In summary, no downscaling approach can produce an absolute result, and none is superior, primarily due to significant method-to-method variability^14^. Hence, this study employs the bilinear interpolation technique for downscaling purposes.

Due to their inherent imperfections, involving conceptualisation, discretisation, and spatial averaging within the grid cells, GCMs are subject to systematic error and bias^15,16^. Recent studies discuss on several methods for bias correction^15,28^, among which the empirical quantile mapping technique is used in this research, namely the “qmap”^29^ package in R programming^22^. Specifically, quantile mapping is the process of establishing a transfer function to harmonise the quantiles of GCM variables with those of observed variables, thus eliminating any bias in the distribution of the variable. In this study, Eqs. (1), (2), (3), and (4) are used for mapping historical precipitation and temperature data with observed values to obtain the bias-corrected data.

1 $$P_{his} \left( d \right)^{*} = F_{obs,m}^{ - 1} \left\{ {F_{his,m} \left( {P_{his,m} } \right)} \right\}$$

2 $$P_{sim} \left( d \right)^{*} = F_{obs,m}^{ - 1} \left\{ {F_{his,m} \left( {P_{his,m} } \right)} \right\}$$

3 $$T_{his} \left( d \right)^{*} = F_{obs,m}^{ - 1} \left\{ {F_{his,m} \left( {T_{m } } \right)} \right\}$$

4 $$T_{sim} \left( d \right)^{*} = F_{obs,m}^{ - 1} \left\{ {F_{his,m} \left( {T_{m } } \right)} \right\}$$

where $P$ and $T$ refer to precipitation and temperature. Subscripts $his$, $obs,$ and $sim$ indicate the historical, observed, and simulated data of corresponding variables while $d$, $m,$ and $*$ refer to the daily, monthly, and corrected data of the respective variables. Moreover, $F$ refers to the cumulative distribution function (CDF) and $F^{ - 1}$ is its inverse.

### Hydrological modelling

The SWAT^17^ is a physically based, continuous, semi-distributed model originally developed to assess the impact of management practices on water, sediment, and agricultural chemical yields in large ungauged basins. Over the past two decades, it has been extensively used to study hydrological issues in conjunction with climate change and management practices^9,30–32^. Since the model functions over the fundamentally reliable principle of water-balance Eq. (5), it has proven its performance over a wide range of basin scales and geo-climatic conditions^33–35^. Hence, it has been chosen for hydrological simulation during the investigation. Detailed information regarding the SWAT model can be accessed through the ‘Soil and Water Assessment Tool Theoretical Documentation Version 2009’^36^.

5 $$SW_{t} = SW_{0} + \mathop \sum \limits_{i = 1}^{t} \left( {R_{day} - Q_{surf} - E_{a} - w_{seep} - Q_{gw} } \right)$$

where $SW_{t}$ = final soil water content (mm) at time step t (days), $SW_{0}$ = initial soil water content (mm), $R_{day}$ = amount of precipitation on ith day (mm),$Q_{surf}$ = amount of surface runoff on ith day (mm), $E_{a}$ = amount of evapotranspiration on ith day (mm), $w_{seep}$ = amount of percolation and bypass flow exiting the soil profile bottom on ith day (mm), $Q_{gw}$ = amount of return flow on ith day (mm).

A schematic of the SWAT model setup, consisting of calibration and validation is embedded within Supplementary Figure 7. To sustain uniformity in the hydrological modelling process among the selected river basins, identical approaches are employed. However, changes in the calibration and validation period are inevitable due to data availability. Details of the fitted parameters and calibration and validation period are provided in the results section along with model performance (see Supplementary Tables 2 and 3). Sensitivity analysis and model calibration are conducted simultaneously on a daily time scale to identify key parameters, evaluated in accordance with their influence over model output for better process representation. Calibration is executed employing the SUFI-2 algorithm in a computer program called “SWAT-CUP”. Subsequently, model performance is evaluated based upon three statistical indicators, namely Nash–Sutcliffe efficiency (NSE), root mean square error to standard deviation of observed discharge (RSR), and percentage bias^18^. In addition, a hydrograph is used to visualise the fitness between simulated and observed flow regime (see Supplementary Fig. 1), statistically calculated using the coefficient of determination. Supplementary Table 4 presents the model performance evaluation criteria employed in this study.

### Projection of future hydrology and hydrological extremes

The calibrated SWAT model is supplied with bias-corrected future climate data to simulate the future hydrology of all river basins under study. The results from the calibrated model are used to analyse the change between baseline and simulated flow under different future climate scenarios. During the study, the computer program Indicators of Hydrologic Alteration (IHA)^37^ version 7.1 is used to analyse the altered hydrological regimes. Changes in the annual and seasonal flow regime are used to assess the impact of climate change on hydrology, whereas changes in Q5 (flow exceeding 5% of the considered analysis period) and Q95 (flow exceeding 95% of the considered analysis period) are used to assess the impact of climate change on hydrological extremes.

## Supplementary Information

Supplementary Information.
