# Supplementary material for: Future hydrology and hydrological extremes under climate change in Asian river basins
Source: Sci Rep. 2021 Aug 24;11:17089. doi: 10.1038/s41598-021-96656-2 (PMC8385063; doi:10.1038/s41598-021-96656-2)
Supplement: Supplementary file 1 — Supplementary Information. [file 41598_2021_96656_MOESM1_ESM.pdf]

Future Hydrology and Hydrological Extremes under Climate Change in Asian River Basins

Sangam Shrestha[1,*], Deg-Hyo Bae[2], Panha Hok[1], Suwas Ghimire[1], and Yadu Pokhrel[3]

[1]Water Engineering and Management, School of Engineering and Technology, Asian Institute of Technology, P.O. Box 4 Klong Luang, Pathum Thani 12120, Thailand

[2]Department of Civil and Environmental Engineering, Sejong University, 98 Gunja-dong, Gwangjin-gu, Seoul, 143–747, Korea

[3]Department of Civil and Environmental Engineering, Michigan State University, East Lansing, MI USA 48824

*e-mail: sangam@ait.ac.th; sangamshrestha@gmail.com

Supplementary Tables

Supplementary Table 1 | Performance evaluation of GCMs' climate simulation

Note: pr: precipitation, tasmax: maximum temperature, tasmin: minimum temperature, rlut: top of atmosphere (TOA) outgoing longwave radiation, psl: sea level pressure, tos: sea surface temperature (SST), ta: air temperature, tas: near-surface air temperature, zg: geopotential height, hus: specific humidity, ua: eastward (zonal) wind, va: northward (meridional) wind. Variables labeled '*' are based on geopotential height (1000, 850, 700, 500, 250, 100, 50, 10 hectopascal (hPa) in 3-D group utilizing 850hPa data

GCMs	Score of each variable												Total score	Rank	Select (O, X)
	pr	tasmax	tasmin	rlut	psl	tos	Ta*	tas	Zg*	Hus*	Ua*	Va*			
ACCESS1.0	1	1	0	1	-1	0	1	-1	-1	-1	1	1	2	8	X
ACCESS1.3	-1	-1	1	0	-1	0	1	-1	-1	-1	1	1	-2	13	X
bcc-csm1-1	-1	-1	-1	-1	1	0	-1	1	1	1	0	0	-1	12	X
BCC-bcc-csm1-1-m	1	1	1	1	1	0	1	1	1	1	-1	1	9	2	O
BNU-ESM	1	-1	-1	1	1	1	-1	1	1	1	-1	0	3	7	X
CCCma-CanESM2	1	-1	-1	1	-1	1	1	1	1	1	1	1	6	5	O
CMCC-CM	-1	1	1	0	-1	0	1	1	1	1	1	1	6	6	X
CMCC-CMCC-CMS	1	1	1	0	-1	0	1	1	1	1	1	1	8	4	O
CNRM-CERFACS-CNRM-CM5	1	1	-1	1	1	0	1	1	1	1	1	1	9	3	O
GFDL-CM3	1	1	1	1	-1	0	1	-1	-1	-1	1	0	2	9	X
GFDL-ESM2G	0	1	-1	1	-1	0	-1	-1	-1	-1	1	1	-2	14	X
HadGEM2-CC	1	1	1	1	-1	1	-1	-1	-1	-1	-1	1	0	11	X
IPSL-CM5A-LR	-1	0	1	-1	0	0	1	-1	-1	-1	-1	-1	-5	16	X
IPSL-CM5A-MR	-1	1	1	-1	0	0	1	-1	-1	-1	0	-1	-3	15	X
IPSL-CM5B-LR	-1	-1	-1	-1	0	0	-1	-1	-1	-1	-1	-1	-10	19	X
MIROC-ESM	-1	-1	-1	0	0	1	-1	-1	-1	-1	-1	-1	-8	18	X
MIROC-ESM-CHEM	-1	-1	-1	1	0	1	-1	-1	-1	-1	-1	-1	-7	17	X
MRI-CGCM3	-1	0	1	-1	1	1	1	-1	-1	-1	1	1	1	10	X
NCC-NorESM1-M	0	1	1	1	1	1	1	1	1	1	1	0	10	1	O

Supplementary Table 2 | Calibrated parameters of SWAT model

South Asian River basins										Southeast Asian River basins									
**Parameters**	**Swat**	**Satpara**	**West Seti**	**Budhi Gandaki**	**Upper Tamakosi**	**Indrawati**	**Tamor**	**Bagmati**	**Wangchu**	**Chindwin**	**Nan**	**Sangkhram**	**Bago**	**Yang**	**Sekong**	**Lamchi**	**Sesan**	**Srepok**	**Bandung**
V_CH_K2.rte	56.1								113.6	7.9	240.1	307.5	2.4	402.8	10.4	347.8	18.9	9.1	1.7
V_GW_REVAP.gw	0.1	0.1	0.0	0.1	0.1		0.1	0.1	0.1		0.1	0.0			0.1		0.1	0.2	0.1
V_EPCO.hRu	0.4											0.3	0.8		0.0		0.3	0.6	0.2
V_CANMX.hRu	5.9	8.9	8.6	7.7	0.6		7.3	6.7	0.1			9.1	16.6		1.7		4.8	1.9	0.4
R_SOL_AWC().sol	0.1					0.2			0.4			0.2	0.4		0.2	0.7	0.1	0.2	-0.2
V_GWQMN.gw	406.4								533.0			523.9	224.9		332.7		286.1	463.3	362.4
R_SOL_K().sol	0.3	0.1	-0.1	0.0		0.2	0.1	-0.1	0.5			-0.2	-0.2		0.2		0.2	0.2	-0.3
V_GW_DELAY.gw	27.2	515.2	139.7	202.5	11.8		202.6	237.3	199.2	28.4	14.5	7.6	173.2	0.0	18.3	8.6	1.5	78.9	46.0
V_OV_N.hRu	5.7	22.5	11.5	6.5	22.6		13.1	4.4				9.6	17.1		24.9		20.5	22.7	16.9
R_SOL_ALB().sol	0.0								-0.2			0.0	-0.1		0.1		0.3	0.3	0.0
V_ALPHA_BF.gw	0.0	0.5	0.2	0.2	0.4		0.4	0.7	0.1			0.8	0.2		0.1	0.4	0.0	0.6	0.4
V_CN2.mgt	-0.4	0.2	-0.2	0.0	-0.4	-0.3	0.0	-0.4	0.4	0.2	0.4	-0.2	0.0	-0.5	0.2	-0.6	0.5	0.1	0.1
V_REVAPMN.gw		183.2	118.2	76.3	171.5	1.4	58.5	101.4	532.3			243.0	392.2		355.7		283.2	264.9	261.2
V_ESCO.hru	0.3	0.6	0.5	0.1	0.7	0.9	0.6	0.4	0.9		0.4	0.6	0.3		1.0	0.9	1.0	0.5	0.5
V_CH_N2.rte	0.3	0.1	0.1	0.3	0.2		0.1	0.1	0.2	0.3	0.2	0.0	0.1	0.2	0.3	0.1	0.2	0.1	0.0
V_RCHRG_DP.gw						0.0				0.3	0.4	0.0	0.2	0.1		0.1			
V_ALPHA_BNK.rte	0.7	0.9	0.3	1.0	0.6	0.4	0.4	0.4		0.6		0.3	0.5	0.8		0.5		0.8	
V_CH_K1.sub										3.8	191.8			227.7					
V_TRNSRCH.bsn										0.2	0.0			0.2		0.1			0.3
V_PLAPS.sub		-4.4	50.6	492.4	14.5		0.7	-163.6				56.0							
V_TLAPS.sub		0.8	-6.0	-4.6	1.5		1.2	0.0				4.8							

Parameters	South Asian River basins									Southeast Asian River basins									
	**Swat**	**Satpara**	**West Seti**	**Budhi Gandaki**	**Upper Tamakosi**	**Indrawati**	**Tamor**	**Bagmati**	**Wangchu**	**Chindwin**	**Nan**	**Sangkhram**	**Bago**	**Yang**	**Sekong**	**Lamchi**	**Sesan**	**Srepok**	**Bandung**
V_SLSUBBSN.hru	72.3	84.5	24.8	11.5	53.3	2.6	17.6	35.7				82.8	145.5	144.4					104.7
V_CH_S1.sub		2.0	3.1	3.8	3.6	0.1	1.3	6.8											
V_HRU_SLP.hru	0.4	0.0	0.6	0.7	1.0	1.0	0.6	0.5					0.4						0.1
V_TIMP.bsn		0.4	0.4	0.4	0.6		0.4	0.8											
V_SNOCOVMX.bsn		398.8	309.5	467.2	51.1		54.0	339.6											
R_SOL_ALB(..).sol		-0.3	0.0	0.2	-0.1		0.1	0.0											
V_SMFMX.bsn		6.7	2.6	0.1	5.4		6.6	7.7											
V_CH_N1.sub		12.8	10.3	8.9	5.2		16.7	8.5											
V_LAT_TTIME.hru	41.6	22.8	9.2	6.8	178.6		11.3	7.6	36.3			17.2	33.4	135.0		93.8		1.7	17.1
V_CH_L1.sub						3.1													
R_SOL_Z(..).sol	0.1					-0.6					0.1		0.3						
V_SURLAG.bsn	3.4											15.0	17.6			20.8		19.6	8.3
V_SHALLST.gw											7483.3								
R_SOL_BD(..).sol									0.2										

Supplementary Table 3 | Statistical performance of SWAT model during calibration (C) and validation (V) periods

SN	Country	Calibration and Validation (Daily)			Performance metrics			
		**Basin**	**Station**	**Period**	**NSE**	**R^2**	**RSR**	**PBIAS (%)**
1	Pakistan	Swat	Chakdara	C: 1984-1997	0.66	0.68	0.59	12.82
				V: 1998-2009	0.34	0.64	0.82	43.08
2	Pakistan	Satpara	Satpara	C: 1990-2000	0.56	0.59	0.66	1.39
				V: 2000-2005	0.47	0.54	0.73	35.45
3	Nepal	West Seti	Gopaghat	C: 1999-2005	0.85	0.85	0.39	1.67
				V: 2006-2008	0.85	0.85	0.38	1.73
4	Nepal	Budhi Gandaki	Arughat	C: 1999-2005	0.74	0.83	0.51	22.23
				V: 2006-2008	0.77	0.80	0.48	16.18
5	Nepal	Upper Tamakoshi	Busti	C: 2004-2007	0.80	0.80	0.45	-1.44
				V: 2008	0.73	0.80	0.52	-17.56
6	Nepal	Indrawati	Dolalghat	C: 2006-2008	0.85	0.86	0.39	10.01
				V: 2009	0.88	0.91	0.35	1.46
7	Nepal	Tamor	Majhitar	C: 1999-2005	0.85	0.85	0.39	1.28
				V: 2006-2008	0.86	0.87	0.37	11.50
8	Nepal	Bagmati	Bhorleni	C: 2000-2005	0.71	0.71	0.54	0.89
				V: 2006-2008	0.54	0.56	0.68	-1.89
9	Bhutan	Wangchu	Chimakoti	C: 2009-2012	0.60	0.74	0.63	32.08
				V: 2013	0.27	0.44	0.85	35.68
10	Myanmar	Chindwin	Mawlaik	C: 2000-2009	0.89	0.89	0.34	1.17
				V: 2010-2016	0.87	0.89	0.36	14.12
11	Thailand	Nan	Ban Bun Nak	C: 2000-2008	0.74	0.75	0.51	2.37
				V: 2009-2012	0.67	0.75	0.58	-31.77
12	Thailand	Sangkhram	Ban Pak Un	C: 1992-2005	0.79	0.83	0.45	10.04
				V: 2006-2014	0.80	0.80	0.45	-12.68
13	Myanmar	Bago	Bago	C: 1997-2003	0.73	0.78	0.52	-25.68
				V: 2004-2005	0.85	0.86	0.39	-21.01
14	Thailand	Yang	Ban Kut Kwang	C: 2000-2004	0.82	0.83	0.42	8.53
				V: 2005-2007	0.72	0.75	0.53	0.71
15	Cambodia	Sekong	Siempang	C: 2001-2008	0.74	0.78	0.51	18.79
				V: 2009-2011	0.71	0.77	0.54	15.24
16	Thailand	Lamchi	Buriram Bridge	C: 2001-2008	0.63	0.63	0.61	-5.33
				V: 2009-2012	0.55	0.58	0.67	-35.05
17	Cambodia	Sesan	Voeun Sai	C: 2000-2007	0.74	0.81	0.51	22.67
				V: 2008-2011	0.73	0.79	0.52	18.88
18	Cambodia	Srepok	Lumphat	C: 2000-2007	0.46	0.49	0.73	-6.24
				V: 2008-2010	0.65	0.74	0.59	27.69
19	Indonesia	Bandung	Saguling	C: 2002-2006	0.63	0.63	0.61	9.05
				V: 2007-2008	0.61	0.62	0.63	-1.10

Supplementary Table 4| Model performance evaluation criteria[18]

Performance Rating	NSE	R^2	RSR	PBIAS
Very Good	0.75 - Unity	0.75 - 1.00	0 - 0.5	<±10
Good	0.65 - 0.75	0.65 - 0.75	0.5 - 0.6	±10 - ±15
Satisfactory	0.50 - 0.65	0.50 - 0.65	0.6 - 0.7	±15 - ±25
Unsatisfactory	< 0.50	< 0.50	> 0.7	> ±25

Supplementary Table 5 | Characteristics of selected Asian river basins.

SN	Country	Basin	Basin Centroid		Elevation (m)		Area (km^2)	Rainfall (mm/yr)	Tavg (°C)
			Lat	Lon	Min.	Max.			
South Asia									
1	Pakistan	Swat	35.2	72.4	373	5898	5728	1121	7
2	Pakistan	Satpara	35.1	75.6	2251	5254	284	418	-3
3	Nepal	West Seti	29.6	81.1	590	7036	4322	1831	20
4	Nepal	Budhi Gandaki	28.6	84.8	419	7979	3848	948	16
5	Nepal	Upper Tamakoshi	28.0	86.3	797	7311	2912	2593	14
6	Nepal	Indrawati	27.9	85.6	595	5838	1230	1938	18
7	Nepal	Tamor	27.5	87.8	360	8385	4376	2216	16
8	Nepal	Bagmati	27.4	85.5	52	2941	3587	2022	19
9	Bhutan	Wangchu	27.4	89.5	156	7304	4570	832	11
Southeast Asia									
10	Myanmar	Chindwin	25.6	95.5	87	3823	69924	2776	25
11	Thailand	Nan	18.8	100.9	124	2072	11620	1342	26
12	Thailand	Songkhram	17.6	103.7	52	676	12885	1732	27
13	Myanmar	Bago	17.5	96.3	-8	816	4881	2870	27
14	Thailand	Yang	16.4	104.0	110	595	4117	1357	26
15	Cambodia, Lao PDR & Vietnam	Sekong	14.9	106.8	43	2210	28227	1661	26
16	Thailand	Lamchi	14.7	103.3	110	548	5139	1245	28
17	Vietnam & Cambodia	Sesan	14.2	107.5	42	2409	18394	1581	13
18	Vietnam & Cambodia	Srepok	12.9	107.7	56	2409	30640	2398	15
19	Indonesia	Bandung	-7.0	107.7	634	2598	1816	2230	23

Supplementary Table 6 | Hydro-meteorological data availability in each river basin.

Note: All river basins are calibrated at single discharge station except Sekong River Basin (3 stations), DEM: Digital Elevation Model

Country	Basin	Rainfall data		Temperature data		Discharge data	Soil data	Landuse data	DEM
		Periods	No. of stations	Periods	No. of stations	Duration (Years)			
Pakistan	Swat	1979-2009 (31 yrs)	16	1979-2009 (31 yrs)	16	1984-2009 (26 yrs)	FAO	ESA	ASTER
Pakistan	Satpara	1987-2005 (19 yrs)	1	1987-2005 (19 yrs)	1	1990-2009 (20 yrs)	FAO	ESA	ASTER
Nepal	West Seti	1996-2008 (13 yrs)	5	1996-2008 (13 yrs)	3	1999-2008 (10 yrs)	SOTER	ESA	ASTER
Nepal	Budhi Gandaki	1996-2008 (13 yrs)	4	1996-2008 (13 yrs)	4	1999-2008 (10 yrs)	SOTER	ESA	ASTER
Nepal	Upper Tamakosi	2001-2008 (8 yrs)	3	2001-2008 (8 yrs)	1	2004-2008 (5 yrs)	SOTER	ESA	ASTER
Nepal	Indrawati	2003-2009 (7 yrs)	12	2003-2009 (7 yrs)	2	2006-2009 (4 yrs)	FAO	ESA	ASTER
Nepal	Tamor	1996-2008 (13 yrs)	6	1996-2008 (13 yrs)	2	1999-2008 (10 yrs)	SOTER	ESA	ASTER
Nepal	Bagmati	1997-2008 (12 yrs)	25	1997-2008 (12 yrs)	14	2000-2008 (9 yrs)	FAO	ESA	ASTER
Bhutan	Wangchu	2008-2013 (6 yrs)	9	2008-2013 (6 yrs)	5	2009-2013 (5 yrs)	FAO	ESA	ASTER
Myanmar	Chindwin	1997-2016 (20 yrs)	4	1997-2016 (20 yrs)	4	2000-2016 (17 yrs)	FAO	ESA	SRTM
Thailand	Nan	1997-2012 (16 yrs)	12	1997-2012 (16 yrs)	2	2000-2012 (13 yrs)	FAO	LDD	ASTER
Thailand	Songkhram	1989-2014 (26 yrs)	30	1989-2014 (26 yrs)	6	1992-2014 (23 yrs)	FAO	LDD	ASTER
Myanmar	Bago	1993-2005 (13 yrs)	4	1993-2005 (13 yrs)	4	1997-2005 (9 yrs)	FAO	ESA	ASTER
Thailand	Yang	1995-2007 (13 yrs)	14	1995-2007 (13 yrs)	6	2000-2007 (8 yrs)	FAO	LDD	ASTER
Vietnam, Laos Cambodia	Sekong	1998-2011 (14 yrs)	4	1998-2011 (14 yrs)	6	2001-2011 (11 yrs)	MRC	MRC	SRTM
Thailand	Lamchi	1996-2012 (17 yrs)	12	1996-2012 (17 yrs)	4	2001-2012 (12 yrs)	FAO	LDD	ASTER
Vietnam & Cambodia	Sesan	1997-2011 (15 yrs)	5	1997-2011 (15 yrs)	6	2000-2011 (12 yrs)	MRC	MRC	SRTM
Vietnam & Cambodia	Srepok	1997-2010 (14 yrs)	7	1997-2010 (14 yrs)	6	2000-2010 (11 yrs)	MRC	MRC	SRTM
Indonesia	Bandung	2001-2008 (8 yrs)	8	2001-2008 (8 yrs)	1	2002-2008 (7 yrs)	FAO	ESA	ASTER

Supplementary Figures

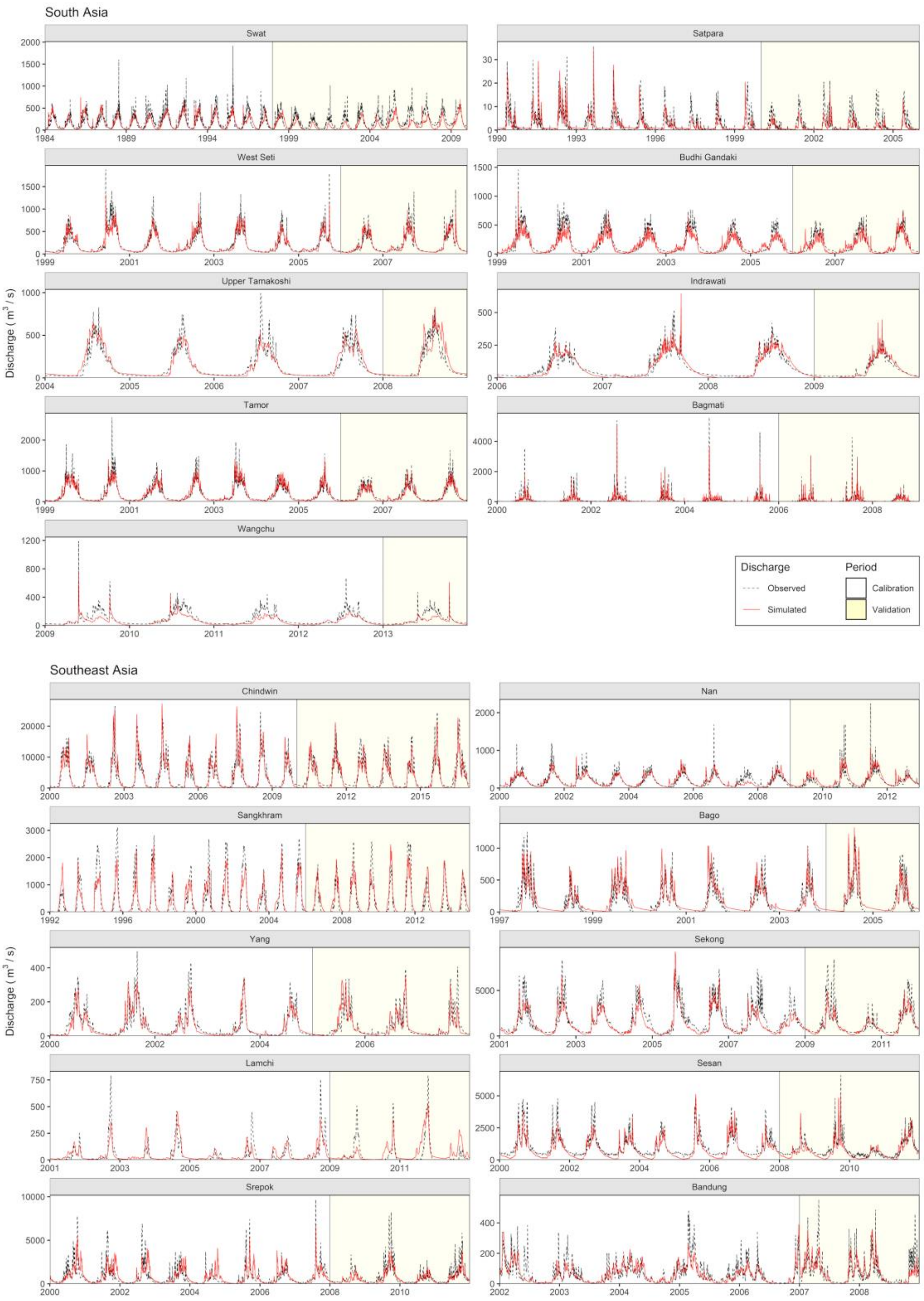

Supplementary Figure 1 | Comparison between observed and simulated flow of South & Southeast Asian river basins. The white and light-yellow area represent calibration and validation period respectively[20,22].

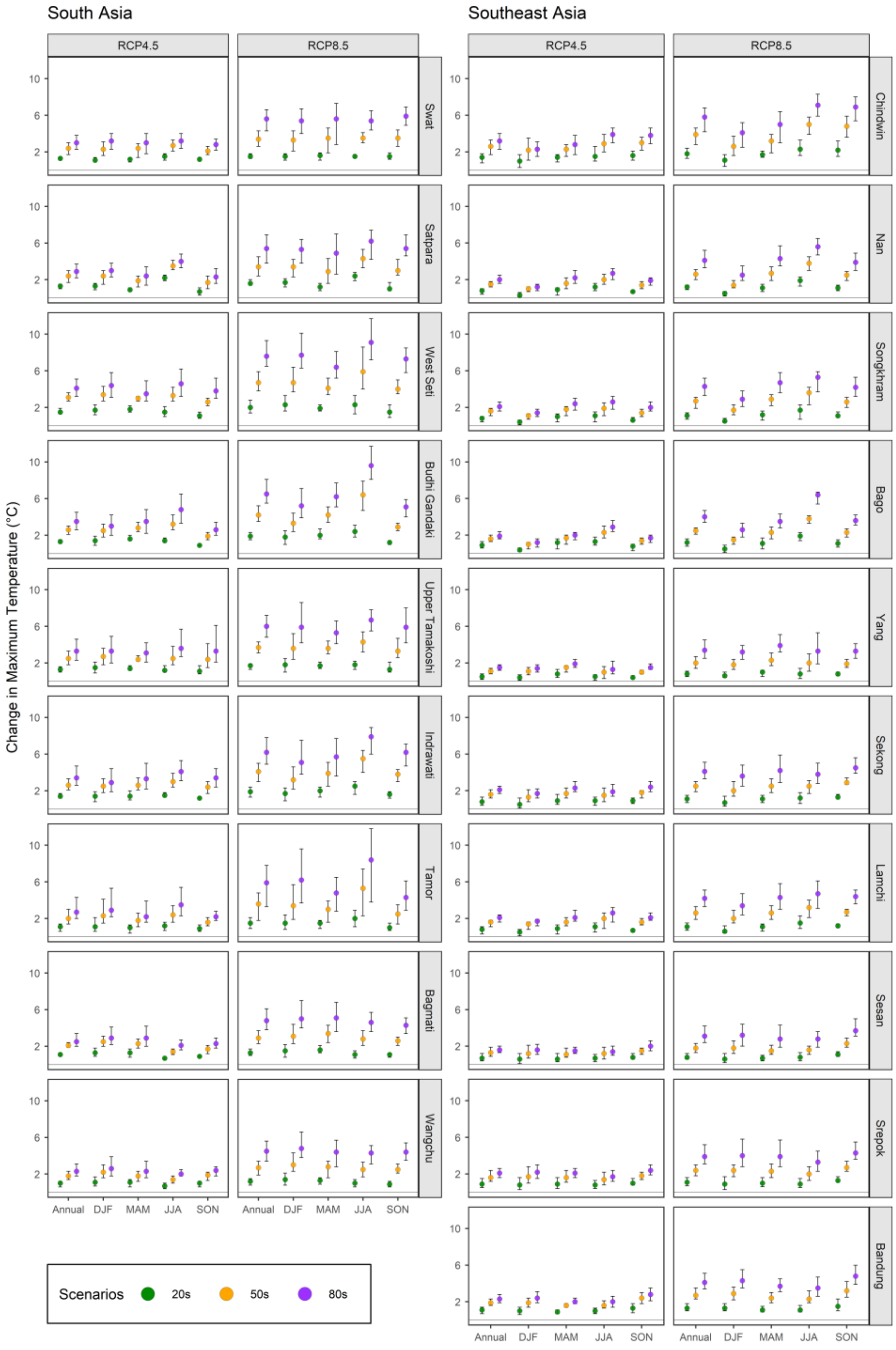

Supplementary Figure 2 | Annual and seasonal maximum temperature (°C) anomaly (absolute change) in South and Southeast Asian River Basins. The results are from an ensemble of GCMs, with each facet corresponding to a different emission scenario (RCP4.5 or RCP8.5) from different basins as indicated at the top and left side of the chart, respectively. Error bar represents the minimum and maximum value among GCMs while the point represents the mean value[20,22].

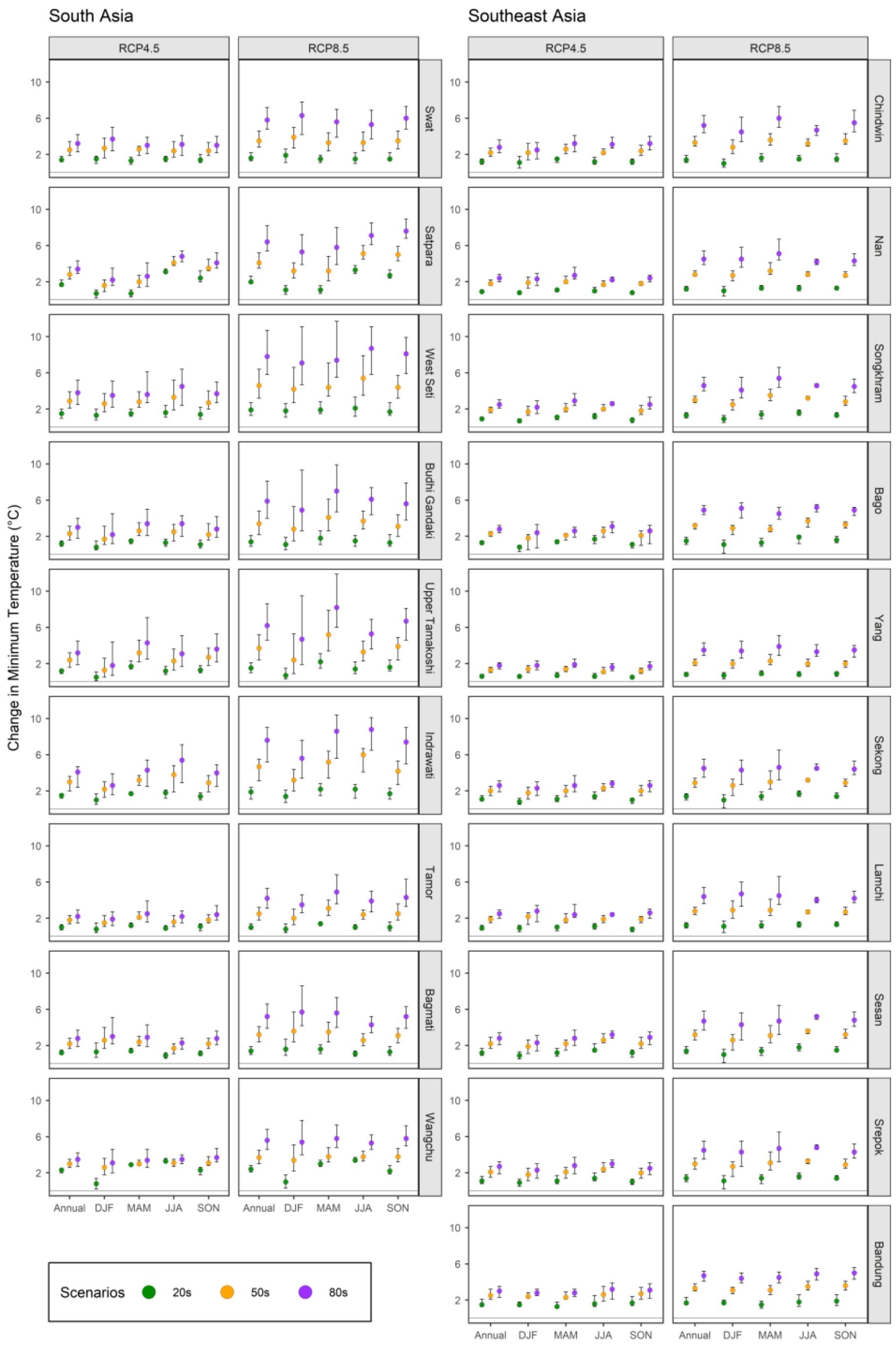

Supplementary Figure 3 | Annual and seasonal minimum temperature (°C) anomaly (absolute change) in South and Southeast Asian River Basins. The results are from an ensemble of GCMs, with each facet corresponding to a different emission scenario (RCP4.5 or RCP8.5) from different basins as indicated at the top and left side of the chart, respectively. Error bar represents the minimum and maximum value among GCMs while the point represents the mean value[20,22].

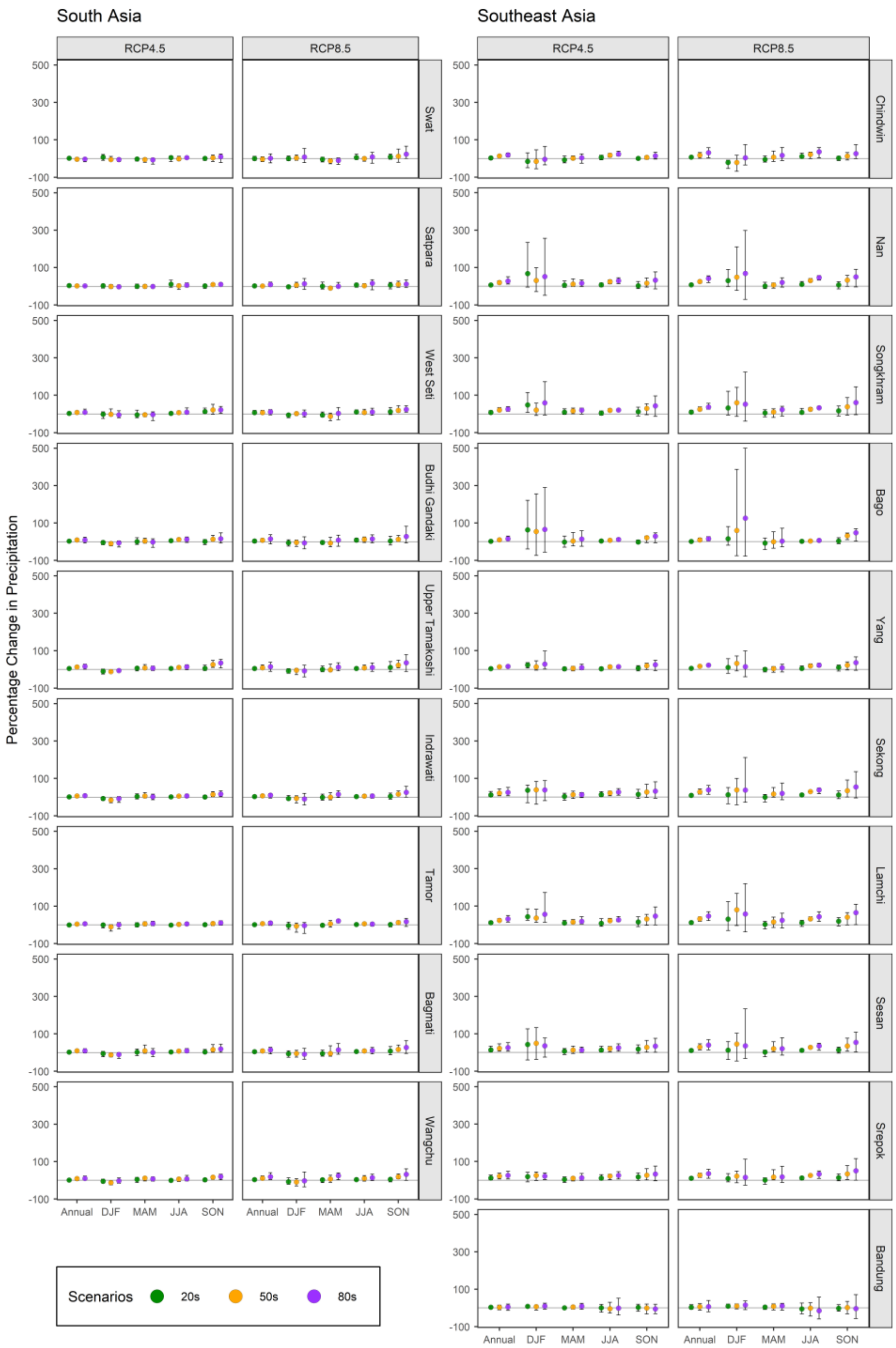

Supplementary Figure 4 | Annual and seasonal precipitation anomaly (% change) in South and Southeast Asian River Basins. The results are from an ensemble of GCMs, with each facet corresponding to a different emission scenario (RCP4.5 or RCP8.5) from different basins as indicated at the top and left side of the chart, respectively. Error bar represents the minimum and maximum value among GCMs while the point represents the mean value[20,22].

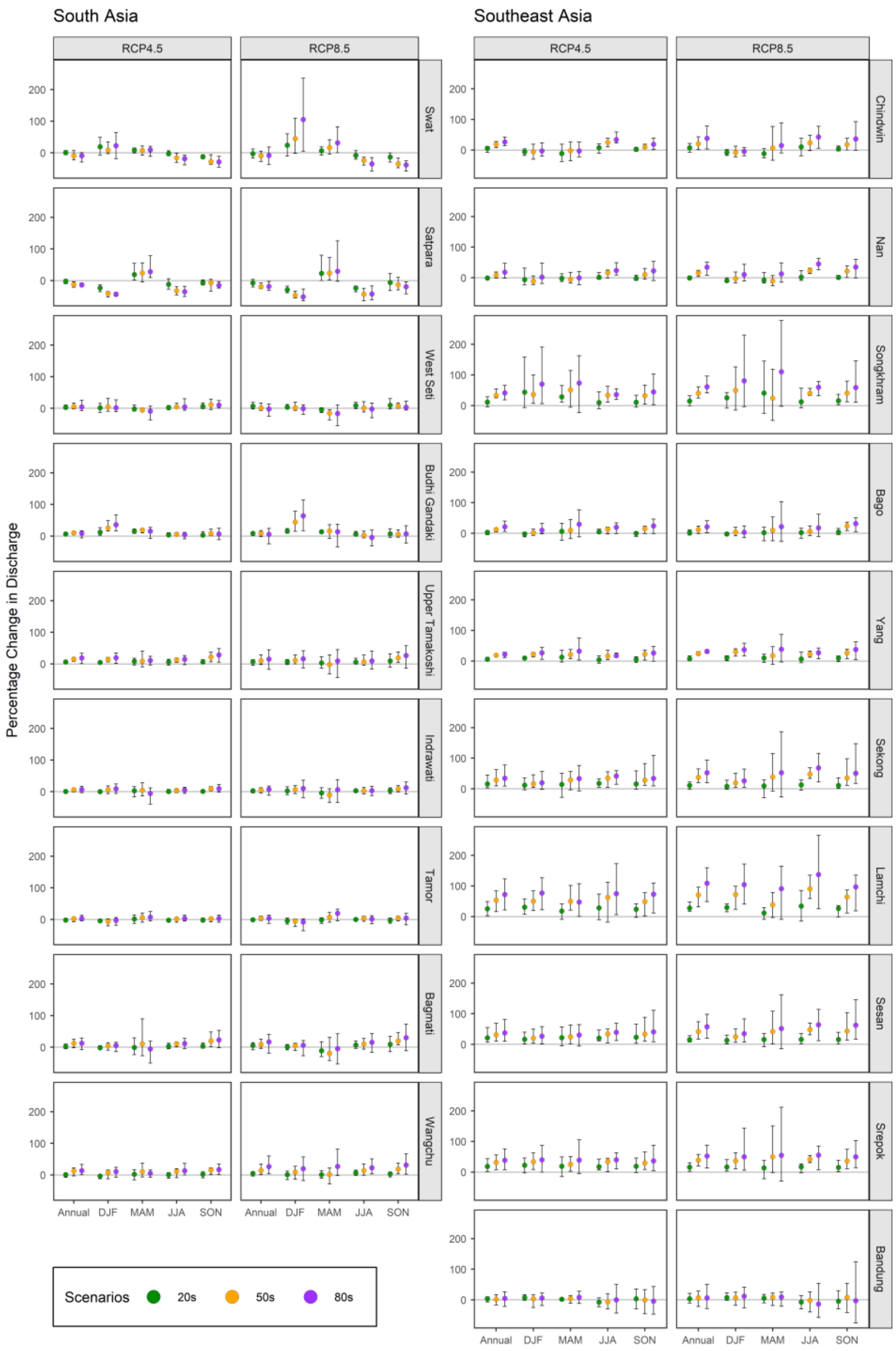

Supplementary Figure 5 | Annual and seasonal discharge anomaly (% change) in South and Southeast Asian River Basins. The results are from an ensemble of GCMs, with each facet corresponding to a different emission scenario (RCP4.5 or RCP8.5) from different basins as indicated at the top and left side of the chart, respectively. Error bar represents the minimum and maximum value among GCMs while the point represents the mean value[20,22].

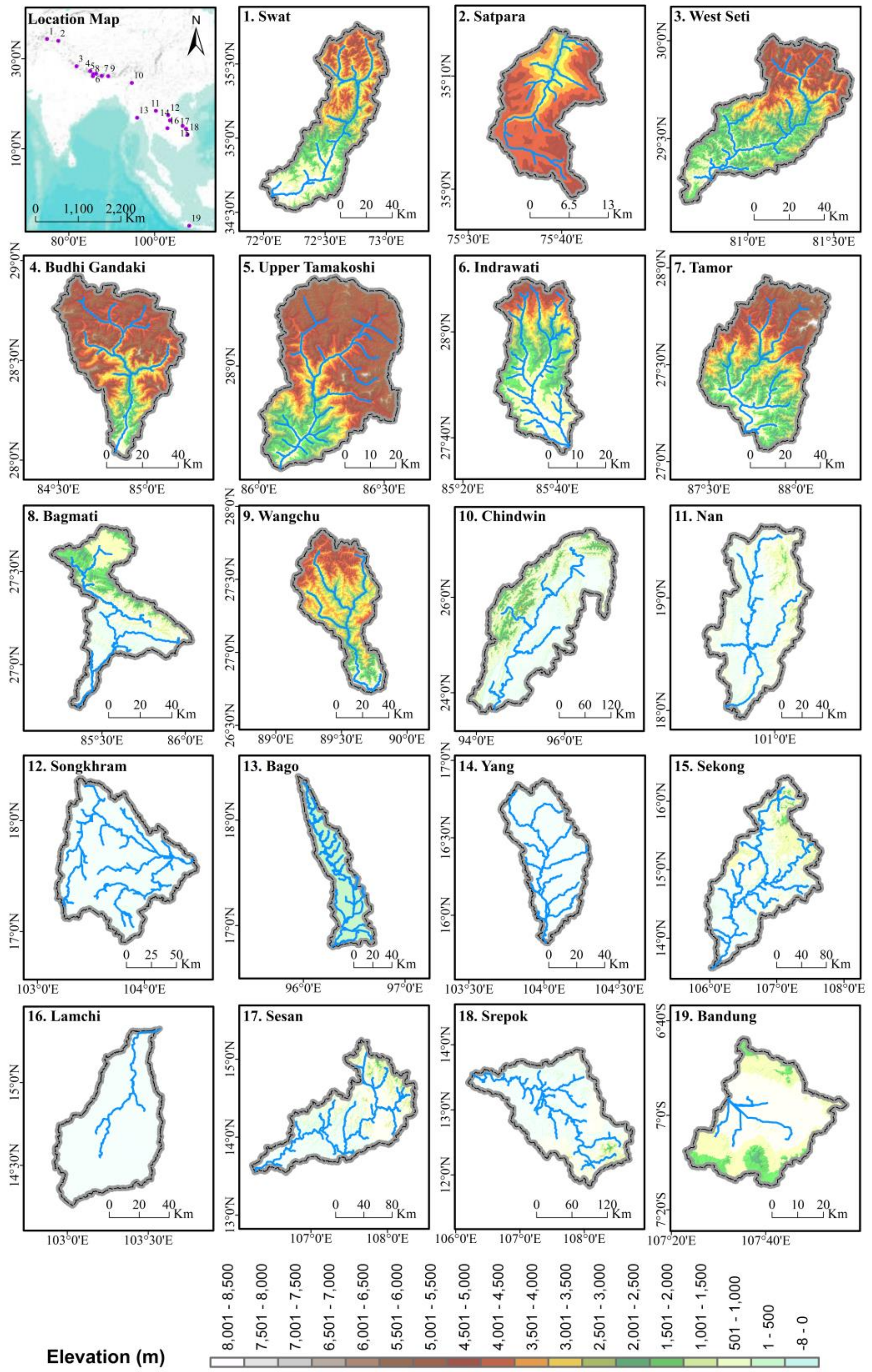

Supplementary Figure 6 | Location map and selected study basins from South and Southeast Asia[38].

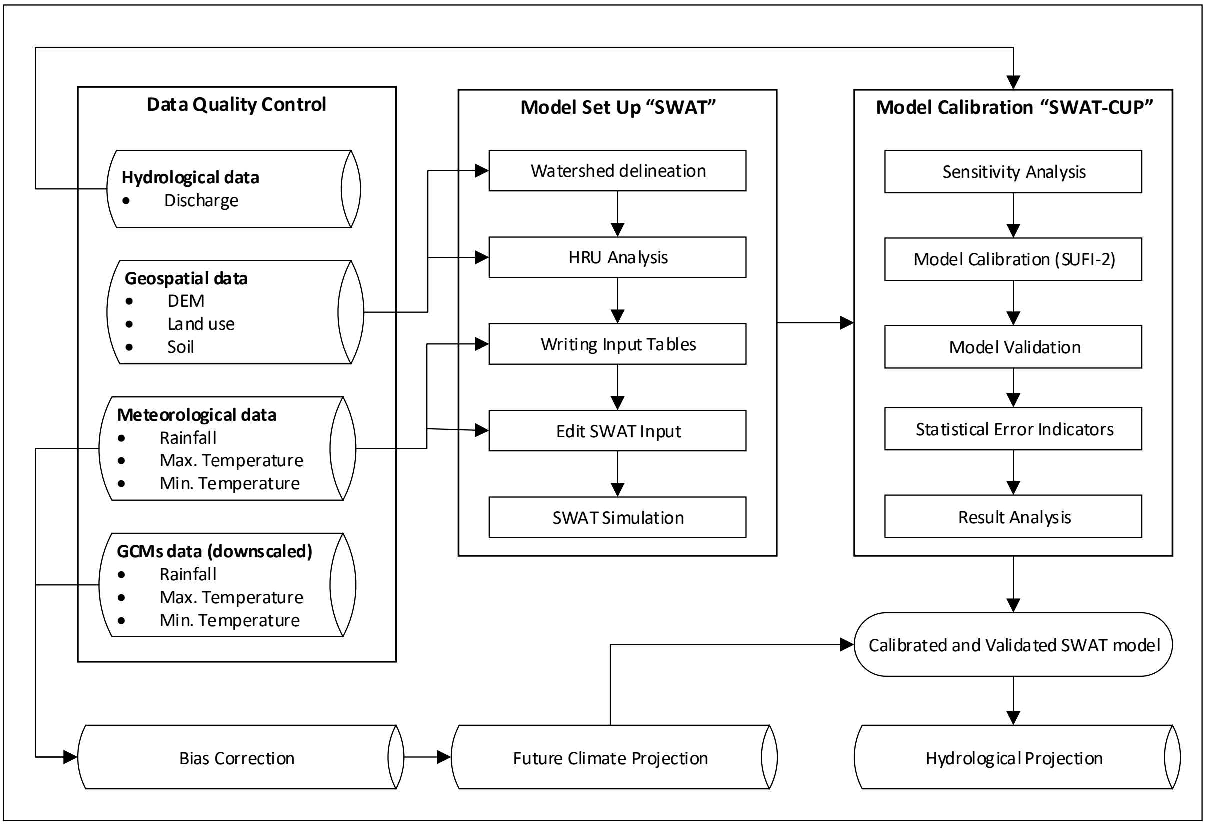

Supplementary Figure 7 | Overall research framework for climate and hydrological projection
